# Supplementary material for: Protein aggregates are associated with replicative aging without compromising protein quality control
Source: eLife. 2015 Nov 6;4:e06197. doi: 10.7554/eLife.06197 (PMC4635334; doi:10.7554/eLife.06197)
Supplement: Supplementary file 1. — Yeast strains used in this study. DOI: http://dx.doi.org/10.7554/eLife.06197.015 [file elife06197s001.docx]

| **Strain** | **Genotype** |
| --- | --- |
| yYB 5644 | MATa ade2::hisG his3 leu2 lys2 ura3D0 trp1D63 hoD::SCW11pr-Cre-EBD78-NatMX loxP-UBC9-loxP-LEU2 loxP-CDC20-Intron-loxP-HPHMX |
| yYB5658 | MATalpha ade2::hisG his3 leu2 met15D::ADE2 ura3D0 trp1D63 hoD::SCW11pr-Cre-EBD78-NatMX loxP-UBC9-loxP-LEU2 loxP-CDC20-Intron-loxP-HPHMX HSP104-Cherry:KanMX |
| yYB5695 | MATa ade2::hisG his3 leu2 met15D::ADE2 ura3D0 trp1D63 hoD::SCW11pr-Cre-EBD78-NatMX loxP-UBC9-loxP-LEU2 loxP-CDC20-Intron-loxP-HPHMX Dcp2-GFP:HIS3 HSP104-Cherry:KanMX |
| yYB5762 | MATa ade2::hisG his3 leu2 met15D::ADE2 ura3D0 trp1D63 hoD::SCW11pr-Cre-EBD78-NatMX loxP-UBC9-loxP-LEU2 loxP-CDC20-Intron-loxP-HPHMX Cdc10-GFP:HIS3 HSP104-Cherry:KanMX |
| yYB5841 | MATa ade2::hisG his3 leu2 met15D::ADE2 ura3D0 trp1D63 hoD::SCW11pr-Cre-EBD78-NatMX loxP-UBC9-loxP-LEU2 loxP-CDC20-Intron-loxP-HPHMX SSA1-GFP:HIS3 HSP104-Cherry:KanMX |
| yYB11439 | MATa ade2::hisG his3 leu2 lys2 ura3D0 trp1D63 hoD::SCW11pr-Cre-EBD78-NatMX loxP-UBC9-loxP-LEU2 loxP-CDC20-Intron-loxP-HPHMX |
| yYB11400 | MATa/alpha ade2::hisG his3 leu2 lys2 ura3D0 trp1D63 hoD::SCW11pr-Cre-EBD78-NatMX loxP-UBC9-loxP-LEU2 loxP-CDC20-Intron-loxP-HPHMX Hsp104-GFP:HIS3/Hsp104-GFP:HIS3 |
| yYB11401 | MATa ade2::hisG his3 leu2 met15D::ADE2 ura3D0 trp1D63 hoD::SCW11pr-Cre-EBD78-NatMX loxP-UBC9-loxP-LEU2 loxP-CDC20-Intron-loxP-HPHMX SSA2-GFP:HIS3 HSP104-Cherry:KanMX |
| yYB11402 | MATalpha ade2::hisG his3 leu2 lys2 ura3D0 trp1D63 hoD::SCW11pr-Cre-EBD78-NatMX loxP-UBC9-loxP-LEU2 Sis1-GFP:HIS3 Hsp104-Cherry::kanMX |
| yYB11403 | MATa ade2::hisG his3 leu2 lys2 ura3D0 trp1D63 hoD::SCW11pr-Cre-EBD78-NatMX loxP-UBC9-loxP-LEU2 Ydj1-GFP:HIS3 Hsp104-Cherry::kanMX |
| yYB11404 | MATalpha ade2::hisG his3 leu2 lys2 ura3D0 trp1D63 hoD::SCW11pr-Cre-EBD78-NatMX loxP-UBC9-loxP-LEU2 Hsp26-GFP:HIS3 Hsp104-Cherry::kanMX |
| yYB11405 | MATa ade2::hisG his3 leu2 met15D::ADE2 ura3D0 trp1D63 hoD::SCW11pr-Cre-EBD78-NatMX loxP-UBC9-loxP-LEU2 loxP-CDC20-Intron-loxP-HPHMX HSP42-GFP:HIS3 HSP104-Cherry:KanMX |
| yYB11406 | MATa ade2::hisG his3 leu2 met15D::ADE2 ura3D0 trp1D63 hoD::SCW11pr-Cre-EBD78-NatMX loxP-UBC9-loxP-LEU2 loxP-CDC20-Intron-loxP-HPHMX Hsp82-GFP:HIS3 HSP104-Cherry:KanMX |
| yYB11407 | MATa leu2-3,112 trp1-1 can1-100 ura3-1 ade2-1 his3-11,15 Hsp104-YFP:KanMX |
| yYB11408 | MATa ade2::hisG his3 leu2 met15D::ADE2 ura3D0 trp1D63 hoD::SCW11pr-Cre-EBD78-NatMX loxP-UBC9-loxP-LEU2 loxP-CDC20-Intron-loxP-HPHMX SSA1-GFP:HIS3 |
| yYB11409 | MATa ade2::hisG his3 leu2 met15D::ADE2 ura3D0 trp1D63 hoD::SCW11pr-Cre-EBD78-NatMX loxP-UBC9-loxP-LEU2 loxP-CDC20-Intron-loxP-HPHMX SSA1-GFP:HIS3 hsp104::KanMX |
| yYB11410 | MATa ade2::hisG his3 leu2 met15D::ADE2 ura3D0 trp1D63 hoD::SCW11pr-Cre-EBD78-NatMX loxP-UBC9-loxP-LEU2 loxP-CDC20-Intron-loxP-HPHMX SSA1-GFP:HIS3 hsp42::KanMX |
| yYB11411 | MATalpha ade2::hisG his3 leu2 met15D::ADE2 ura3D0 trp1D63 hoD::SCW11pr-Cre-EBD78-NatMX loxP-UBC9-loxP-LEU2 loxP-CDC20-Intron-loxP-HPHMX SSA1-GFP:HIS3 hsp42::KanMX |
| yYB11412 | MATa ade2::hisG his3 leu2 lys2 ura3D0 trp1D63 hoD::SCW11pr-Cre-EBD78-NatMX loxP-UBC9-loxP-LEU2 loxP-CDC20-Intron-loxP-HPHMX HSP104-GFP:HIS3 hsp42::kanMX |
| yYB11413 | MATa ade2::hisG his3 leu2 lys2 ura3D0 trp1D63 hoD::SCW11pr-Cre-EBD78-NatMX loxP-UBC9-loxP-LEU2 loxP-CDC20-Intron-loxP-HPHMX hsp42::kanMX |
| yYB11414 | MATa ade2::hisG his3 leu2 lys2 ura3D0 trp1D63 hoD::SCW11pr-Cre-EBD78-NatMX loxP-UBC9-loxP-LEU2 loxP-CDC20-Intron-loxP-HPHMX HSP104-GFP:HIS3 hsp26::kanMX |
| yYB11415 | MATa ade2::hisG his3 leu2 lys2 ura3D0 trp1D63 hoD::SCW11pr-Cre-EBD78-NatMX loxP-UBC9-loxP-LEU2 loxP-CDC20-Intron-loxP-HPHMX HSP104-GFP:HIS3 ssa1::kanMX |
| yYB11416 | MATa ade2::hisG his3 leu2 lys2 ura3D0 trp1D63 hoD::SCW11pr-Cre-EBD78-NatMX loxP-UBC9-loxP-LEU2 loxP-CDC20-Intron-loxP-HPHMX HSP104-GFP:HIS3 ssa2::kanMX |
| yYB11417 | MATa ade2::hisG his3 leu2 lys2 ura3D0 trp1D63 hoD::SCW11pr-Cre-EBD78-NatMX loxP-UBC9-loxP-LEU2 loxP-CDC20-Intron-loxP-HPHMX hsp104::kanMX |
| yYB11418 | MATa ade2::hisG his3 leu2 lys2 ura3D0 trp1D63 hoD::SCW11pr-Cre-EBD78-NatMX loxP-UBC9-loxP-LEU2 loxP-CDC20-Intron-loxP-HPHMX hsp104::KanMX hsp42 ::KanMX |
| yYB11419 | MATa his3D1 leu2D0 met15D0 ura3D0 Hsp104-GFP:HIS3 |
| yYB11420 | MATa l his3D1 leu2D0 met15D0 ura3D0 Hsp104-GFP:HIS3 pAG415GPD-Hsp104-mCherry:Leu |
| yYB11421 | MATa his3D1 leu2D0 met15D0 ura3D0 Hsp104-GFP:HIS3 pAG415GPD-Hsp42-mCherry:Leu |
| yYB11422 | MATa his3D1 leu2D0 met15D0 ura3D0 Hsp104-mCherry:KanMX |
| yYB11530 | MATa/MATα ADE2/ade2-101 ADE1/ade1-4 his3/ his3-11 leu2/leu2-3 ura3D0/ura3-1 trp1D63/trp1-1 hoD::SCW11pr-Cre-EBD78-NatMX loxP-UBC9-loxP-LEU2 loxP-CDC20-Intron-loxP-HPHMX Sup35-GFP:HIS3/+ HSP104-mCherry:KanMX/+ [PIN+] [PSI+] |
| yYB11531 | MATa/MATα ADE2/ade2-101 ADE1/ade1-4 his3/ his3-11 leu2/leu2-3 ura3D0/ura3-1 trp1D63/trp1-1 hoD::SCW11pr-Cre-EBD78-NatMX loxP-UBC9-loxP-LEU2 loxP-CDC20-Intron-loxP-HPHMX Sup35-GFP:HIS3/+ HSP104-mCherry:KanMX/+ [PIN+] [psi-] |
| yYB11532 | MATa/MATalpha ade2::hisG/ade2::hisG his3/his3 leu2/leu2 lys2/lys2 ura3D0/ura3D0 trp1D63/trp1D63 hoD::SCW11pr-Cre-EBD78-NatMX/ hoD::SCW11pr-Cre-EBD78-NatMX loxP-UBC9-loxP-LEU2/loxP-UBC9-loxP-LEU2 loxP-CDC20-Intron-loxP-HPHMX/loxP-CDC20-Intron-loxP-HPHMX Hsp104-mCherry-KanMX/Hsp104-GFP-HIS |
| yYB12876 | MATa ade2::hisG his3 leu2 met15D::ADE2 ura3D0 trp1D63 hoD::SCW11pr-Cre-EBD78-NatMX loxP-UBC9-loxP-LEU2 loxP-CDC20-Intron-loxP-HPHMX HSP104-Cherry:KanMX pADH_OsTIR-9myc:TRP pADHeGFP_IAA17_NLS:URA |
| yYB12877 | MATa ade2::hisG his3 leu2 met15D::ADE2 ura3-1 trp1-1 hoD::SCW11pr-Cre-EBD78-NatMX loxP-UBC9-loxP-LEU2 loxP-CDC20-Intron-loxP-HPHMX HSP104-Cherry:KanMX pADH_OsTIR-9myc:URA3 pADH1_eGFP_IAA17:TRP1 |
| yYB12878 | MATa leu2D0 His3D1 ura3D0 HSP104-Cherry:KanMX pESC-URA-Rnq1-GFP |
| yYB12879 | MATa leu2D0 His3D1 ura3D0 HSP104-Cherry:KanMX pESC-LEU-GFP-VHL |
| yYB12880 | MATa ade2::hisG his3 leu2 met15D::ADE2 ADE1-14 ura3D0 trp1D63 hoD::SCW11pr-Cre-EBD78-NatMX loxP-UBC9-loxP-LEU2 loxP-CDC20-Intron-loxP-HPHMX RNQ1-GFP:HIS3 HSP104-Cherry:KanMX [PIN+] [PSI+] |
| yYB12881 | MATa ade2::hisG his3 leu2 lys2 ura3D0 trp1D63 hoD::SCW11pr-Cre-EBD78-NatMX loxP-UBC9-loxP-LEU2 loxP-CDC20-Intron-loxP-HPHMX BTN2-GFP:HIS3 HSP104-Cherry:KanMX |
| yYB12882 | MATa ade2::hisG his3 leu2 met15D::ADE2 ura3D0 trp1D63 hoD::SCW11pr-Cre-EBD78-NatMX loxP-UBC9-loxP-LEU2 loxP-CDC20-Intron-loxP-HPHMX HSP82-GFP:HIS3 HSP104-Cherry:KanMX, rpn4::KanMX |
